# Study of the Mechanism Underlying the Onset of Diabetic Xeroderma Focusing on an Aquaporin-3 in a Streptozotocin-Induced Diabetic Mouse Model

**DOI:** 10.3390/ijms20153782

**Published:** 2019-08-02

**Authors:** Nobutomo Ikarashi, Nanaho Mizukami, Risako Kon, Miho Kaneko, Ryogo Uchino, Izumi Fujisawa, Natsuko Fukuda, Hiroyasu Sakai, Junzo Kamei

**Affiliations:** Department of Biomolecular Pharmacology, Hoshi University, 2-4-41 Ebara, Shinagawa-ku, Tokyo 142-8501, Japan

**Keywords:** aquaporin, diabetes, skin, xeroderma, streptozotocin

## Abstract

Xeroderma is a frequent complication in diabetic patients. In this study, we investigated the mechanism underlying the onset of diabetic xeroderma, focusing on aquaporin-3 (AQP3), which plays an important role in water transport in the skin. Dermal water content in diabetic mice was significantly lower than that in control mice. The expression level of AQP3 in the skin was significantly lower in diabetic mice than in control mice. One week after streptozotocin (STZ) treatment, despite their increased blood glucose levels, mice showed no changes in the expression levels of AQP3, *Bmal1*, *Clock*, and *D site-binding protein* (*Dbp*) in the skin and 8-hydroxydeoxyguanosine (8-OHdG) in the urine. In contrast, two weeks after STZ treatment, mice showed increases in the blood glucose level, decreases in AQP3, *Bmal1*, *Clock*, and *Dbp* levels, and increases in the urinary levels of 8-OHdG. The results of this study suggest that skin AQP3 expression decreases in diabetes, which may limit water transport from the vessel side to the corneum side, causing dry skin. In addition, in diabetic mice, increased oxidative stress triggered decreases in the expression levels of *Bmal1* and *Clock* in the skin, thereby inhibiting the transcription of *Aqp3* by *Dbp*, which resulted in decreased AQP3 expression.

## 1. Introduction

The number of diabetic patients continues to increase with changes in lifestyle and social environments [1]. In diabetes, subjective symptoms such as polyuria and thirst occur as initial symptoms, and nephropathy, retinopathy, and peripheral neuropathy develop as the disease progresses further. In addition to these complications, many patients also develop various skin diseases, and the proportion of these patients is approximately 80% in clinical practice [2,3,4]. Xeroderma is characterized by dryness of the skin due to loss of moisture and occurs in 1 out of 4 diabetic patients. The prevention and treatment of xeroderma are important because this condition not only reduces patient quality of life but can also cause other skin diseases, such as skin infections and gangrene. Moisturizing agents are currently used as a symptomatic therapy for xeroderma [3]. However, since diabetic xeroderma occurs throughout the body and moisturizing agents only exert an effect if applied to the affected site several times a day, the convenience and therapeutic effect of these agents cannot be considered sufficient. In addition, although most moisturizing agents contain glycerol as the main ingredient, there is little scientific evidence for the effectiveness of glycerol against xeroderma. Therefore, elucidation of the mechanism underlying the onset of diabetic xeroderma has been attempted for the purposes of prevention and treatment [5], but this mechanism is not yet fully understood.

Recently, it was shown that aquaporins (AQPs) play an important role in water and glycerol transport in the body. AQPs are channels that exist in the cell membrane and allow water and glycerol to pass in and out of the cell according to the osmotic pressure gradient. In humans, 13 subfamilies from AQP0 to AQP12 are distributed throughout the body. AQP3 is abundantly expressed in the skin, and it has been reported that *Aqp3* knockout mice have decreased dermal water content and skin flexibility and elasticity [6]. Recently, the relationships between AQP3 and skin diseases have been highlighted; it has been reported that AQP3 expression decreases during aging [7], psoriasis [8], and vitiligo [9,10] but that AQP3 expression increases during atopic dermatitis [11], scleroderma [12], and skin cancer [13]. During the onset of diabetic xeroderma, water transport in the skin is considered to be an important factor, but the relationship of diabetic xeroderma with skin AQP3 is not well understood. To date, it was known that AQP3 constitutes an important molecule in the onset of diabetes; transgenic *Aqp3*-deficient mice develop nephrogenic diabetes insipidus [14]; changes in the expression of AQP3 in the adipose tissue [15], liver [16], or kidneys [17] have been associated with the development of type 2 diabetes. In this study, we focused on AQP3 in the skin to conduct basic research on the mechanism underlying the onset of diabetic xeroderma, with the aim of proposing new preventive and therapeutic approaches. First, a streptozotocin (STZ)-induced diabetic mouse model was established, and the relationship between the dermal water content and expression level of AQP3 in the skin was analyzed to investigate the role of AQP3 in the development of xeroderma. Then, the mechanism by which the expression level of skin AQP3 varies in diabetes was investigated.

## 2. Results

### 2.1. Dermal Water Content and Transepidermal Water Loss (TEWL)

**Figure 1:**
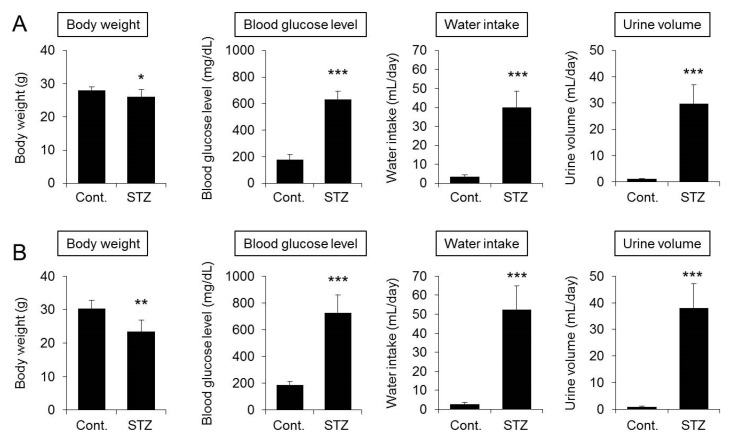
Body weight, blood glucose level, water intake, and urine volume. Mice were given a single intravenous administration of streptozotocin (STZ). After four weeks (**A**) or eight weeks (**B**), body weight, the blood glucose level, water intake, and urine volume were measured (mean ± SD, *n* = 5; * *p* < 0.05, ** *p* < 0.01, and *** *p* < 0.001).

**Figure 2:**
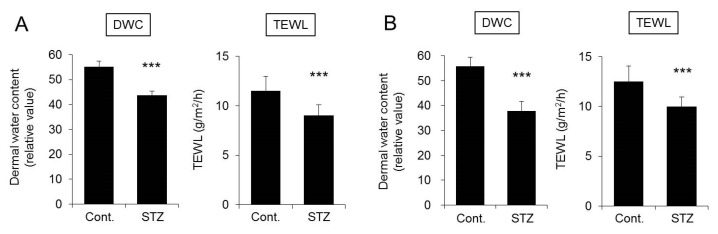
Dermal water content and transepidermal water loss (TEWL). Mice were given a single intravenous administration of STZ. After four weeks (**A**) or eight weeks (**B**), the dermal water content (DWC) and TEWL were measured (mean ± SD, *n* = 5, *** *p* < 0.001).

The body weight was significantly decreased in mice four weeks after STZ treatment. In addition, the blood glucose level, water intake, and urine volume of the STZ-treated group were all significantly higher than those of the control group (Figure 1A). The dermal water content and TEWL of the STZ-treated group were both significantly lower than those of the control group (Figure 2A). The changes in mice at eight weeks after STZ treatment were similar to those observed four weeks after STZ treatment (Figure 1B and Figure 2B).

Based on the above data, it was confirmed that mice treated with STZ exhibited diabetic conditions, including skin dryness.

### 2.2. Expression Levels of AQP3 in the Skin

**Figure 3:**
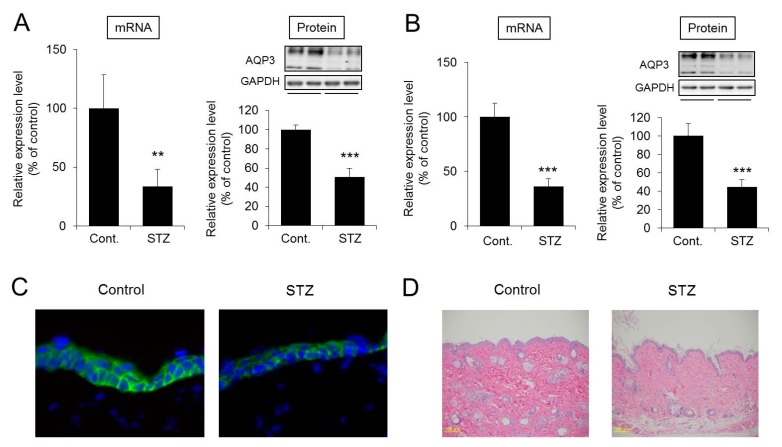
Expression levels and distribution of AQP3 in the skin. Mice were given a single intravenous administration of STZ. After four weeks (**A**) or eight weeks (**B**), the *Aqp3* mRNA expression level in the skin was measured by real-time RT-PCR. After normalization to *18S rRNA*, the data are presented with the mean value of the control group set at 100%. The protein expression of AQP3 in the skin was analyzed by Western blotting. After normalization to glyceraldehyde-3-phosphate dehydrogenase (GAPDH), the data are shown with the mean value of the control group set at 100% (mean ± SD, *n* = 5; ** *p* < 0.01 and *** *p* < 0.001). At four weeks after STZ treatment, AQP3 (green) and nuclei (blue) in mouse skin were immunostained (**C**). Skin tissue was assessed by hematoxylin and eosin (HE) staining (**D**).

The mRNA expression level of *Aqp3* in the skin was significantly lower in the STZ treatment group than in the control group at four weeks. The protein expression level of AQP3 was also significantly lower in the STZ group than in the control group. The results for mice at eight weeks after STZ treatment were similar to those at four weeks after treatment (Figure 3A,B). In the immunohistochemistry, AQP3 was only detected in the stratum basal [18], and it was also confirmed that the density of AQP3 in the skin of the STZ treatment group was lower than that of control group (Figure 3C). No tissue damage was observed in the skin samples from the STZ-treated group (Figure 3D).

These results demonstrate that in diabetes, AQP3 expression is decreased without skin tissue damage.

### 2.3. Relationship between the Blood Glucose Level and Skin AQP3 Expression Level

The results to date show that in diabetes, skin AQP3 expression is decreased, and the skin becomes dry. Thus, the relationship between an increased blood glucose level and a decreased skin AQP3 level was examined to investigate the mechanism underlying the decreased AQP3 expression. Briefly, blood glucose and skin AQP3 expression levels were analyzed in mice at one and two weeks after STZ treatment.

**Figure 4:**
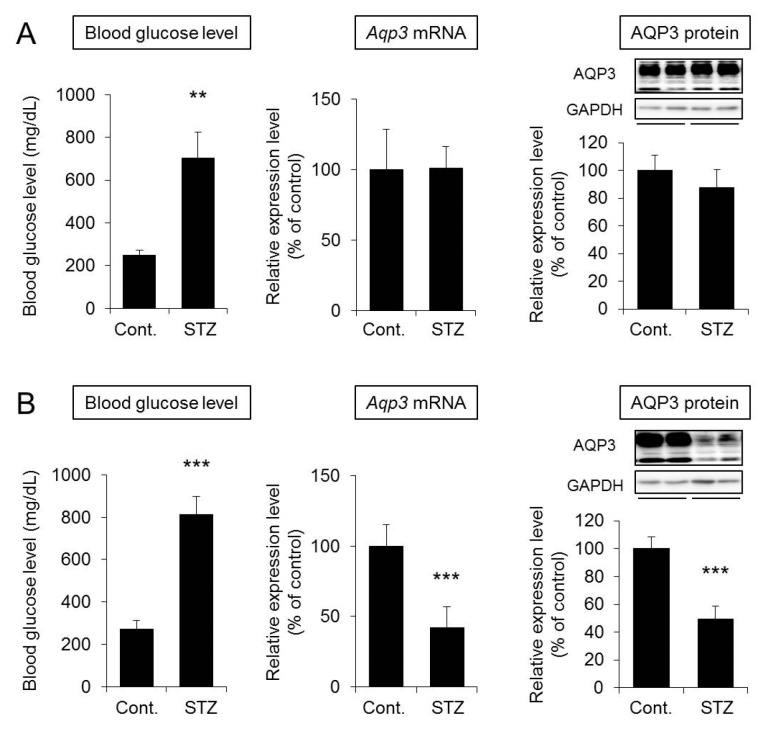
Relationship between the blood glucose level and skin AQP3 level. Mice were given a single intravenous administration of STZ. After one week (**A**) or two weeks (**B**), blood glucose levels were measured. The *Aqp3* mRNA expression level in the skin was analyzed by real-time RT-PCR. After normalization with *18S rRNA*, the data are presented with the mean value of the control group set at 100%. The protein expression of AQP3 in the skin was analyzed by Western blotting. After normalization with GAPDH, the data are shown with the mean value of the control group set at 100% (mean ± SD, *n* = 5; ** *p* < 0.01 and *** *p* < 0.001).

Blood glucose levels were significantly higher in the STZ group than in the control group at one week. However, the mRNA and protein expression levels of AQP3 in the skin were similar between the STZ group and the control group (Figure 4A). In contrast, increased blood glucose and decreased AQP3 expression were observed in mice at two weeks after STZ treatment (Figure 4B). Water intake and urine volume were increased in mice at both one week and two weeks after STZ treatment, confirming the pathological condition of diabetes (Figure S1).

Thus, there was no correlation between the increased blood glucose level and decreased skin AQP3 level.

### 2.4. Expression Levels of Bmal1, Clock, and D Site-Binding Protein (Dbp) in the Skin

It has been reported that circadian rhythms in the whole body are disrupted in diabetes [19,20,21]. In addition, it was recently reported that the expression level of *Aqp3* is regulated by the clock genes *Bmal1* and *Clock* and the downstream signal *Dbp* [22,23]. Therefore, it is possible that the expression levels of *Bmal1* and *Clock* in the skin are decreased in diabetes and that *Aqp3* levels are decreased through the decrease in *Dbp* expression. In this study, the expression levels of *Bmal1*, *Clock*, and *Dbp* in mouse skin were analyzed at one and two weeks after STZ treatment to investigate the relationships with the decrease in *Aqp3* expression.

**Figure 5:**
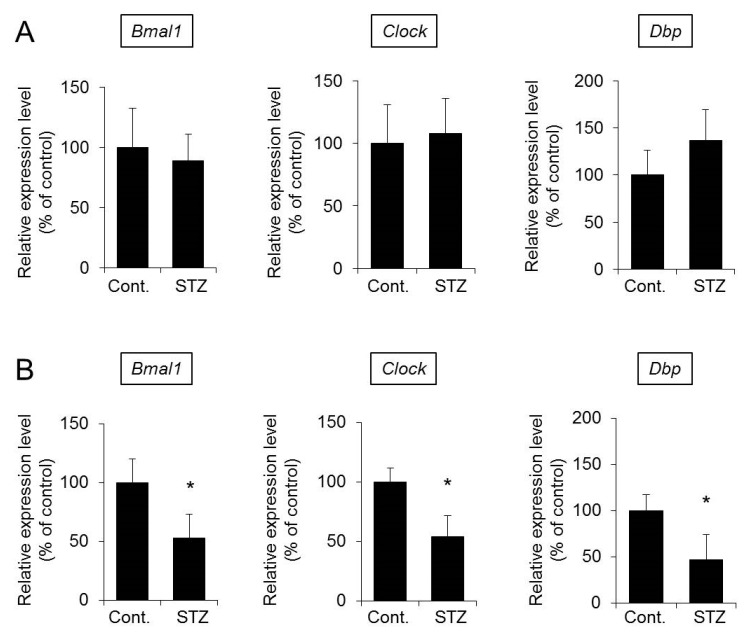
Expression levels of *Bmal1*, *Clock*, and *Dbp* in the skin. Mice were given a single intravenous administration of STZ. After one week (**A**) or two weeks (**B**), the mRNA expression levels of *Bmal1*, *Clock*, and *Dbp* in the skin were measured by real-time RT-PCR. After normalization with *18S rRNA*, the data are presented with the mean value of the control group set at 100% (mean ± SD, *n* = 5, * *p* < 0.05).

The mRNA expression levels of *Bmal1*, *Clock*, and *Dbp* in the skin of mice at one week after STZ treatment were comparable to those in the skin of mice in the control group (Figure 5A). In contrast, at two weeks after STZ treatment, mice showed decreased *Aqp3* expression, and the levels of all proteins were significantly decreased in the STZ group compared with the control group (Figure 5B).

These results suggest that abnormal circadian rhythms may be involved in the decrease in *Aqp3* expression in diabetes.

### 2.5. Urinary 8-Hydroxydeoxyguanosine (8-OHdG) Level

Oxidative stress reportedly increases in diabetes [24]. In addition, oxidative stress is known to be involved in the disruption of the circadian rhythms [25,26]. These findings suggest that increased oxidative stress may be involved in the decrease in clock gene expression in the skin of mice at two weeks after STZ treatment. Urinary 8-OHdG levels are increased in diabetic patients [27] and are used as an indicator of oxidative stress [28,29,30]. Therefore, 8-OHdG levels in mouse urine were measured at one and two weeks after STZ treatment to investigate the relationship between oxidative stress and decreased AQP3 expression.

**Figure 6:**
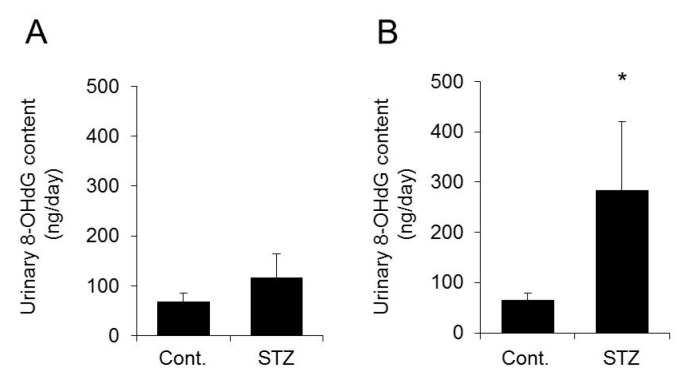
Urinary 8-hydroxydeoxyguanosine (8-OHdG) levels. Mice were given a single intravenous administration of STZ. After one week (**A**) or two weeks (**B**), the 8-OHdG concentration in the urine was measured (mean ± SD, *n* = 5, * *p* < 0.05).

The urinary 8-OHdG level tended to increase at one week after STZ treatment but was not significantly different from the level in the control group (Figure 6A). On the other hand, in mice at two weeks after STZ treatment, which showed a decrease in AQP3 expression, the urinary 8-OHdG level was significantly higher than that in the control mice, with an increase of approximately five-fold (Figure 6B).

Thus, a correlation was found between decreased AQP3 expression and increased oxidative stress.

## 3. Discussion

While many patients with diabetes develop xeroderma, the mechanism of onset is unclear. In this study, we examined whether the water channel AQP3 expressed in the skin is involved in the onset mechanism of diabetic xeroderma.

The STZ-induced type 1 diabetes mouse model was analyzed in this study. STZ administration to mice is associated with symptoms characteristic of type 1 diabetes, including marked weight loss, polydipsia, and polyuria [31,32]. In this study, body weight was decreased, and blood glucose, water intake, and urine volume were increased in mice at four weeks after STZ treatment, confirming the pathological development of diabetes. In STZ-treated mice, the dermal water content was also found to be significantly decreased, confirming that dry skin is induced in the mice as in diabetic patients [3,4]. Analysis of the expression levels of skin AQP3 in mice at four weeks after STZ treatment revealed significant decreases at both the mRNA and protein levels compared with the levels in control group mice. Similarly, increased blood glucose levels, dry skin, and decreased AQP3 expression were observed in mice at eight weeks after STZ treatment. In *Aqp3* knockout mice, the dermal water content has been reported to decrease [6]. Therefore, it is possible that the expression level of AQP3 in the skin decreases in diabetes, the transportation of water from the vessel side to the corneum side is limited, and the dermal water content is decreased. No skin disorder was observed in the STZ-treated mice, and TEWL did not increase, suggesting that dry skin development was not due to reduced barrier function.

Why did the expression of skin AQP3 decrease in diabetes? We first identified the point after STZ treatment at which AQP3 expression decreased. The results showed that skin AQP3 expression was similar in STZ-treated mice and control mice at one week after STZ treatment, although an increased blood glucose level was observed. In contrast, although an increase in the blood glucose level was observed in mice at two weeks after STZ treatment, unlike at one week, skin AQP3 expression was significantly decreased. These results showed that the expression of AQP3 in the skin decreased during the second week after STZ treatment. In addition, it was found that the increase in the blood glucose level was unlikely to cause a decrease in skin AQP3 directly.

It has been reported that *Aqp3* expression can be increased or decreased by various substances [33,34,35,36,37]. Matsunaga et al. recently reported that skin *Aqp3* expression was decreased in *Clock*-deficient mice and that dermal water content was also decreased [22]. In addition, *Bmal1* and *Clock* regulate *Dbp* transcription in mice, and when Dbp binds to D-box, *Aqp3* is transcribed [22]. On the other hand, it has been reported that the addition of hydrogen peroxide to HaCaT cells, a human skin keratinocyte cell line, suppresses the transcriptional activity of *Bmal1* [25]. It has also been reported that increased oxidative stress in various cells decreases the expression of clock genes [26,38]. Given that persistent increases in the blood glucose level lead to increased oxidative stress [24], the decreased expression of skin *Aqp3* in diabetes may be related to a disturbance of circadian rhythms associated with increased oxidative stress. Therefore, this point was analyzed in mice at one and two weeks after STZ treatment. Examination of the levels of urinary 8-OHdG, a systemic oxidative stress marker, revealed no change in urinary 8-OHdG levels in mice that did not show a decrease in *Aqp3* expression at one week after STZ treatment. No differences were observed in the expression levels of *Bmal1*, *Clock*, and *Dbp* in the skin between the STZ group and the control group. In contrast, in mice with decreased *Aqp3* expression at two weeks after STZ treatment, the urinary 8-OHdG level was significantly higher than that in control mice, with an increase of approximately five-fold, and at the same time, the expression levels of *Bmal1*, *Clock*, and *Dbp* were all significantly decreased. These changes correlated with the decrease in *Aqp3* expression. Based on the above data, it was concluded that increased oxidative stress in diabetes triggered decreases in the expression levels of *Bmal1* and *Clock* in the skin, thereby inhibiting the transcription of *Aqp3* by *Dbp*, which resulted in decreased protein expression levels of AQP3. In this study, the extent of locally increased oxidative stress in the skin and types of reactive oxygen species were not analyzed. However, since the expression levels of *superoxide dismutase* (*Sod*) *1* and *Sod2*, which eliminate the reactive oxygen species, superoxide anion, were decreased in the skin in the STZ group compared with the control group (Figure S2), it was thought that reactive oxygen species levels are also increased locally in the skin. AQP3 has been found to mediate membrane hydrogen peroxide uptake, and AQP3-mediated hydrogen peroxide transport is associated with the development of psoriasis [39,40,41,42,43]. Therefore, even in diabetic xeroderma, due to the decrease in skin AQP3, intracellular hydrogen peroxide levels decreases and extracellular hydrogen peroxide levels increases, which may further decrease the expression of clock genes; thereby xeroderma may be getting worse.

It has previously been reported that, in addition to the decreased dermal water content, wound healing is delayed in diabetes [44,45]. Recently, functional analysis of AQP3 has indicated that AQP3 in the skin is involved in cell proliferation and cell migration [46,47], and delayed wound healing has been confirmed in *Aqp3*-deficient mice [6]. Therefore, the decrease in skin AQP3 expression in diabetes may be involved not only in xeroderma but also in delayed wound healing.

AQP3 is an aquaglyceroporin that permeates not only water, but also glycerol, which constitutes an important molecule for skin hydration [48,49,50]. Therefore, it is possible that the glycerol content in the skin of STZ-treated mice was lower than that of control mice. It was known that topic or systemic glycerol administration or AQP3 re-expression in keratinocytes reverts the phenotype of skin abnormalities of *Aqp3*-deficient mice [50,51]. The present results are considered to indicate the scientific evidence of glycerol containing the moisturizing agents used for the treatment of xeroderma.

The results of this study revealed that AQP3 expression decreases at the onset of diabetic xeroderma, which may be associated with increased oxidative stress. Therefore, vitamin D and vitamin E, antioxidants that suppress oxidative stress, were considered useful for the treatment and prevention of diabetic xeroderma. Vitamin A [36] and a peroxisome proliferator-activated receptor (PPAR) γ agonist [37], which increases AQP3 levels, may also be useful for diabetic xeroderma treatment. In the future, antioxidants and substances that increase AQP3 expression are expected to be applied for diabetic xeroderma therapy.

## 4. Materials and Methods

### 4.1. Animals and Treatments

Male HR-1 hairless mice (seven weeks old) were purchased from Japan SLC, Inc. (Shizuoka, Japan). The mice were housed at 24 ± 1 °C and 55 ± 1% humidity with 12 h of light (08:00–20:00). The study was conducted upon approval (approval No. 29–117; 29 June 2017) in accordance with the Hoshi University Guiding Principles for the Care and Use of Laboratory Animals.

After 12 h of fasting, mice were given a single intravenous injection of 150 mg/kg STZ dissolved in a 0.05 mol/L citrate buffer (pH 4.5). Mice with a plasma glucose level greater than 500 mg/dL were used in this study. Normal mice were given a single intravenous injection of the citrate buffer. After administration, water intake and urine volume were measured using metabolic cages. TEWL was measured using the Tewameter TM300 (Courage & Khazaka, Cologne, Germany). The dermal water content was measured using the Corneometer CM825 (Courage & Khazaka). After one week, two weeks, four weeks, and eight weeks, mice were anesthetized with diethyl ether, and a blood sample was collected from the abdominal vena cava using heparin. The skin was removed, frozen in liquid nitrogen, and stored at −80 °C.

### 4.2. Blood and Urine Analyses

Blood samples were centrifuged (1000× *g* for 15 min at 4 °C), and the plasma was stored at −80 °C until assays were performed. Urine samples were centrifuged (1000× *g* for 15 min at 4 °C). Plasma glucose concentrations were enzymatically quantified using the Glucose CII-Test Wako (Wako Pure Chemicals, Osaka, Japan). Urinary 8-OHdG concentrations were measured using a New 8-OHdG Check ELISA kit (Nikken Zairu, Tokyo, Japan).

### 4.3. HE Staining

HE staining was performed by the Biopathology Institute Co., Ltd. (Oita, Japan). Briefly, skin samples isolated from mice were immersed in 10% neutral buffered formalin to fix the tissue. The tissue samples were embedded in paraffin and cut into 5 μm sections mounted on glass slides. The slides were stained with hematoxylin followed by eosin for microscopy. The slides were evaluated by a pathologist in a blinded manner, and the skin damage was assessed.

### 4.4. Real-Time RT-PCR

RNA was extracted from mouse tissue samples using TRI reagent (Sigma-Aldrich Corp., St. Louis, MO, USA). A high-capacity cDNA synthesis kit (Applied Biosystems, Foster City, CA, USA) was used to synthesize cDNA from 1 μg of RNA. Target gene expression was analyzed by RT-PCR using the primers (Hokkaido System Science Co., Ltd., Hokkaido, Japan) listed in Table 1. mRNA gene expression levels were normalized to *18S rRNA* gene expression levels.

### 4.5. Preparation of Samples for Western Blotting

Skin tissue was homogenized in a dissecting buffer (0.3 mol/L sucrose, 25 mmol/L imidazole, 1 mmol/L ethylenediaminetetraacetic acid, 8.5 μmol/L leupeptin, and 1 μmol/L phenylmethylsulfonyl fluoride; pH 7.2) on ice. The homogenate was centrifuged (4000× *g* for 15 min at 4 °C), and the resulting supernatant was centrifuged (200,000× *g* for 60 min at 4 °C). The supernatant was removed, and the dissecting buffer was added to the precipitate. The final homogenate was analyzed by Western blotting [52].

### 4.6. Electrophoresis and Western Blotting

Proteins were separated using sodium dodecyl sulfate-polyacrylamide gel electrophoresis and then transferred to a polyvinylidene difluoride membrane. The proteins in the membrane were probed with primary (rabbit anti-rat AQP3 antibody, 1/500, Alomone Labs, Jerusalem, Israel; mouse anti-rabbit GAPDH antibody, 1/10,000, Merck Millipore, Darmstadt, Germany) and secondary antibodies (donkey anti-rabbit IgG-HRP antibody, 1/5000, Santa Cruz Biotechnology Inc., Santa Cruz, CA, USA; sheep anti-mouse IgG-HRP antibody, 1/10,000, Merck Millipore). The recognized proteins were detected using an ECL prime detection reagent (GE Healthcare, Chicago, IL, USA). The protein immunocomplexes were visualized using a Lumino image analyzer (ImageQuant LAS500 system, GE Healthcare). The protein expression level of AQP3 was normalized to that of GAPDH.

### 4.7. Immunohistochemistry

Skin tissue was postfixed in 4% paraformaldehyde. Tissue samples were embedded in optimum cutting temperature compound (Sakura Finetek, Torrance, CA, USA) and frozen by liquid nitrogen. The frozen blocks were cut into 10 μm sections mounted on glass slides. The sections were incubated with a rabbit anti-rat AQP3 antibody (1/200). The sections were treated with an Alexa Fluor 488-conjugated donkey anti-rabbit IgG antibody (1/200, Thermo Fisher Scientific, Waltham, MA, USA). The slides were covered and observed under a fluorescence microscope.

### 4.8. Statistical Analysis

Numerical data are expressed as the mean ± standard deviation. Significance was examined using Student’s *t*-test for pairs of values. Differences with *p* < 0.05 were considered statistically significant.

## Figures and Tables

**Table 1:** Primer sequences for real-time PCR.

Gene	Forward (5′ to 3′)	Reverse (5′ to 3′)
*Aqp3*	CCTTGTGATGTTTGGCTGTGG	GGAAGCACATTGCGAAGGTC
*Bmal1*	TGCAATGTCCAGGAAGTTAGAT	GTTTGCTTCTGTGTATGGGTTG
*Clock*	TCTATGCTTCCTGGTAACGC	GGTTTCCAGTCCTGTCGAATC
*Dbp*	GAAGGAAAAGGAGCGCAAGG	TATTCCACGTCCCCGAAAGG
*18S rRNA*	GTCTGTGATGCCCTTAGATG	AGCTTATGACCCGCACTTAC

## Supplementary Materials

The following are available online at https://www.mdpi.com/1422-0067/20/15/3782/s1, Figure S1: Water intake and urine volume, Figure S2: Expression levels of *superoxide dismutase* (Sod)1 and Sod2 in the skin.

## Abbreviations

8-OHdG	8-hydroxydeoxyguanosine
AQPs	Aquaporins
Dbp	D site-binding protein
DWC	Dermal water content
GAPDH	Glyceraldehyde-3-phosphate dehydrogenase
PPAR	Peroxisome proliferator-activated receptor
SD	Standard deviation
SOD	Superoxide dismutase
STZ	Streptozotocin
TEWL	Transepidermal water loss
