# Supplementary material for: Study of the Mechanism Underlying the Onset of Diabetic Xeroderma Focusing on an Aquaporin-3 in a Streptozotocin-Induced Diabetic Mouse Model
Source: Int J Mol Sci. 2019 Aug 2;20(15):3782. doi: 10.3390/ijms20153782 (PMC6696158; doi:10.3390/ijms20153782)
Supplement: Supplementary file 1 [file ijms-20-03782-s001.pdf]

**Supplementary date:** Study of the mechanism underlying the onset of diabetic xeroderma focusing on an aquaporin-3 in a streptozotocin-induced diabetic mouse model

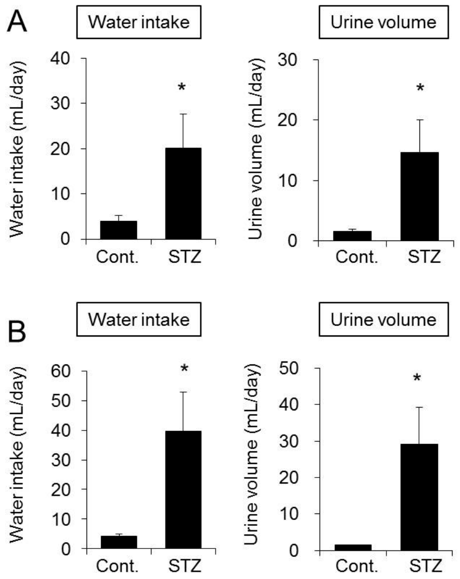


**Figure S1. Water intake and urine volume**

Mice were given a single intravenous administration of STZ. After 1 weeks (A) or 2 weeks (B), water intake and urine volume were measured (mean ± SD, $n = 5$; * $p < 0.05$).

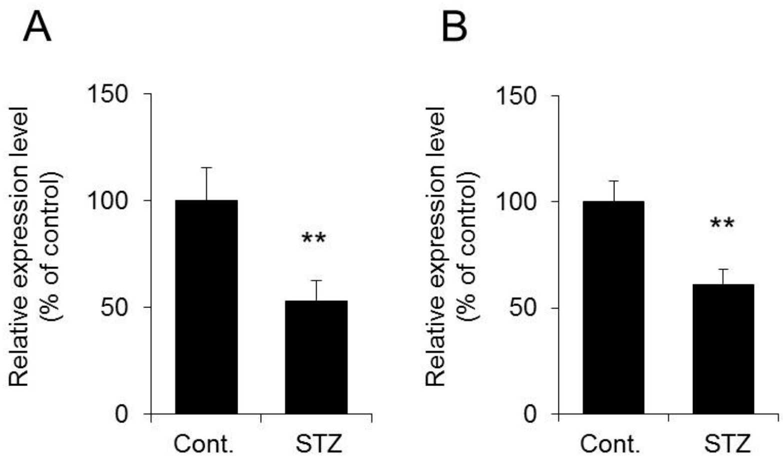


**Figure S2. Expression levels of *Sod1* and *Sod2* in the skin**

Mice were given a single intravenous administration of STZ. After 2 weeks, the mRNA expression levels of *Sod1* and *Sod2* in the skin were measured by real-time RT-PCR. After normalization with *18S rRNA*, the data are presented with the mean value of the control group set at 100% (mean ± SD, $n = 5$, ** $p < 0.01$).
